# Supplementary material for: Initial pseudo-steady state & asymptotic KPZ universality in semiconductor on polymer deposition
Source: Sci Rep. 2017 Jun 19;7:3773. doi: 10.1038/s41598-017-03843-1 (PMC5476714; doi:10.1038/s41598-017-03843-1)
Supplement: Supplementary file 1 — Supplementary Information [file 41598_2017_3843_MOESM1_ESM.pdf]

Supplementary Information for "Initial pseudo-steady state & asymptotic KPZ universality in semiconductor on polymer deposition"

Renan A. L. Almeida[1,*], **Sukarno O. Ferreira**[2], **Isnard Ferraz**[2], **and Tiago J. Oliveira**[2,†]

[1]Tokyo Institute of Technology, Department of Physics, 2-12-1 Ookayama, Meguro-ku, Tokyo, 152-8551, Japan
[2]Departamento de Física, Universidade Federal de Viçosa, 36570-900, Viçosa, Minas Gerais, Brazil
*lisboa.r.aa@m.titech.ac.jp
†tiago@ufv.br

1 X-Ray diffraction measurements

We used a D8-Discover (BRUKER) X-Ray diffractometer (XRD) in the $\theta - 2\theta$ coupled mode (radiation $\lambda_{CuK\alpha} = 0.15418$ nm) for studying CdTe crystalline structure as deposition proceeds. From the XRD spectra (not shown), we calculated the probability of finding grains in the [111] direction, relative to all other allowed directions, as:

$$P([111],t) = \frac{I_{111}(t)/A_{\theta-2\theta(\theta_{111})}}{\sum_{hkl} I_{hkl}(t)/A_{\theta-2\theta}(\theta_{hkl})} \tag{1}$$

where I_{hkl} is the intensity of the peak hkl, and $A_{\theta-2\theta}(\theta_{hkl})$ is the absorption factor for the $\theta - 2\theta$ geometry, dependent on θ_{hkl} angle[1]. Figure S1 shows the temporal evolution of probabilities $P([111],t)$ for both CdTe grown on Kapton and on Si(001) substrates at same conditions. On both substrates, there is an increasing of $P([111],t)$ with t, which indicates the existence of a preferential growth (i.e. a texture) in $[111]$ direction. However, the curves are rather different for short deposition times, with $P([111],t)$ significantly smaller for Kapton substrates. In short, (111)-grains take longer time to dominate the film structure in the Kapton case, probably due to the non-crystalline nature of the polymer. As discussed in the main text, this difference is the key to explain the existence of the pseudo-steady state (PSS) regime for Kapton.

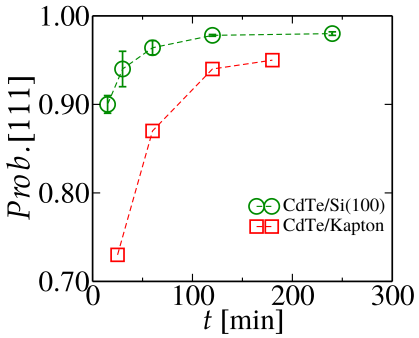

Suppl. Fig. 1. Probability of finding a CdTe grain in the [111] direction as a function of time. Data for CdTe/Si(100) is the same reported in[2].

2 Additional data for width and extremal height distributions

In Fig. 2(d) of the main text we compare width distributions (rescaled to zero mean and unitary variance) with the log-normal distribution (LND). A more complete analysis can be done by comparing distributions rescaled to unitary mean $P(x)$, with $x \equiv \frac{w_2}{\langle w_2 \rangle}$, which are expected[3] to be given by the LND:

$$P(x,t) = \frac{1}{\sqrt{2\pi}\sigma x} \exp\left\{ -\frac{[\ln(x)-\mu]^2}{2\sigma^2} \right\}, \tag{2}$$

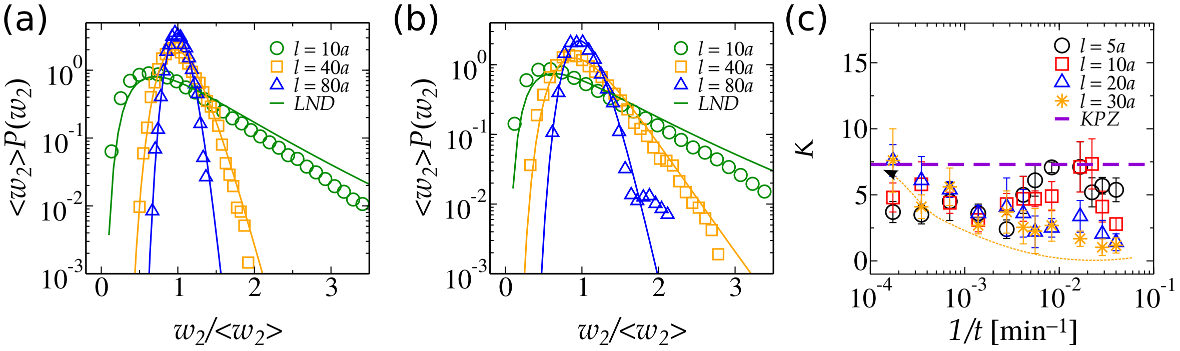

Suppl. Fig. 2. Rescaled width distributions $P(w_2)$ (symbols) for CdTe surfaces for (a) $t = 120$ min (PSS regime) and (b) $t = 720$ min (KPZ regime). Solid lines are LNDs with the respective (experimentally calculated) parameters μ and σ. (c) Kurtosis (K) of $P(w_2)$ as a function of $1/t$ for several window sizes.

when the correlation length ξ is much smaller than the window size l ($\xi \ll l$). In the equation above, $\mu(t) \equiv \langle \ln(x) \rangle$ and $\sigma(t) \equiv \sqrt{\langle \ln^2(x) \rangle - \langle \ln(x) \rangle^2}$. We rescaled distributions in such way, for fixed times in PSS and KPZ regimes and different l's [see Figs. S2(a) and S2(b)]. Indeed, in both regimes we observe a nice agreement between the width distributions and their respective LNDs (with μ and σ obtained directly from the experimental data). As expected, as larger l is, narrower the distributions are, since they are converging to a delta function. Moreover, in the limit of small l the collapses worsen, since the condition $\xi \ll l$ is obviously not satisfied.

The convergence of the kurtosis K for the stationary width distribution (for $\xi \gg l$) is presented in Fig. S2(c). Similarly to the cumulant ratios R and S (shown in Figs. 4(a) and 4(b) of the main text, respectively), we observe that K converges to the KPZ value as t and l becomes large (with $\xi \gg l$).

Figure S3 shows cumulant ratios for (local) maximal height distributions $P(m)$ calculated in the stationary regime ($\xi \gg l$). The skewness is in striking agreement with the value expected[3] for KPZ class, presenting small time- and l-corrections. Values of K for large t and l also agree quite well with the KPZ one. On the other hand, the ratio $R \equiv \langle m \rangle_c / \langle m^2 \rangle_c^{1/2}$ is still rather different from the KPZ value, even for large t and l. One way for dealing with this slow convergence is to take the R values for the longest times and extrapolate them for $l \to \infty$. Performing such procedure [see the inset of Fig. S3(a)], we find $R = 7.3(5)$, which is basically the same R value obtained from simulations of KPZ models[3].

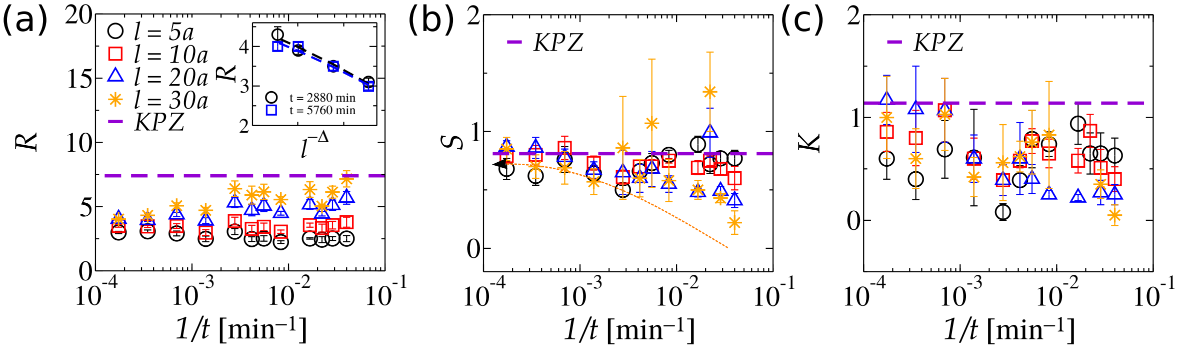

Suppl. Fig. 3. Cumulant ratios (a) R, (b) skewness S and (c) kurtosis K for maximal height distributions as functions of $1/t$, for several window sizes ($l \ll \xi$). The inset in (b) shows a extrapolation of R as $l^{-\Delta}$ goes to zero, where $\Delta = 0.17$ is used to linearize the data.

References

1. Birkholz, M., *Thin film analysis by X-ray scattering* (John Wiley & Sons, 2006).
2. Almeida, R. A. L., Ferreira, S. O., Ribeiro, I. R. B. & Oliveira, T. J. Temperature effect on $(2+1)$ experimental Kardar-Parisi-Zhang growth. *Europhys. Lett.* **109**, 46003 (2015).

3. Carrasco, I. S. S. & Oliveira, T. J. Width and extremal height distributions of fluctuating interfaces with window boundary conditions. *Phys. Rev. E* **93**, 012801 (2016).
